# Tumor Microenvironment in the Brain

**DOI:** 10.3390/cancers4010218

**Published:** 2012-02-22

**Authors:** Mihaela Lorger

**Affiliations:** Leeds Institute of Molecular Medicine, University of Leeds, St. James’s University Hospital, Beckett Street, Leeds, LS9 7TF, UK; E-Mail: M.Lorger@leeds.ac.uk; Tel.: +44-113-3438449; Fax: +44-113-2429886

**Keywords:** tumor microenvironment, brain metastases, brain tumors, microglia, macrophages, astrocytes, pericytes, angiogenesis

## Abstract

In addition to malignant cancer cells, tumors contain a variety of different stromal cells that constitute the tumor microenvironment. Some of these cell types provide crucial support for tumor growth, while others have been suggested to actually inhibit tumor progression. The composition of tumor microenvironment varies depending on the tumor site. The brain in particular consists of numerous specialized cell types such as microglia, astrocytes, and brain endothelial cells. In addition to these brain-resident cells, primary and metastatic brain tumors have also been shown to be infiltrated by different populations of bone marrow-derived cells. The role of different cell types that constitute tumor microenvironment in the progression of brain malignancies is only poorly understood. Tumor microenvironment has been shown to be a promising therapeutic target and diagnostic marker in extracranial malignancies. A better understanding of tumor microenvironment in the brain would therefore be expected to contribute to the development of improved therapies for brain tumors that are urgently required due to a poor availability of treatments for these malignancies. This review summarizes some of the known interactions between brain tumors and different stromal cells, and also discusses potential therapeutic approaches within this context.

## 1. Introduction

Central nervous system (CNS) malignancies include primary brain tumors that originate within the brain, and brain metastases which are initiated upon the dissemination of cancer cells from the primary tumors outside the CNS. Brain metastases develop in 10–25% of cancer patients with extracranial malignancies and account for more than half of all brain tumors in adults. The most common sources of brain metastases are lung cancer, breast cancer, and melanoma. The incidence of brain metastases in the clinic is further increasing, probably due to the improved treatments for primary tumors and extracranial metastases. Metastatic brain lesions tend to develop late in the course of a progressive metastatic disease and often after the development of metastases at extracranial sites. It has been reported that brain is increasingly the sole site of tumor relapse after the initial therapy. Despite recent improvements in the treatment of brain metastases, the median survival time of patients with metastatic brain lesions is only 7–16 months [1,2,3,4].

Gliomas are the most common primary brain tumors and include a variety of histologic and molecular types. According to the World Health Organization (WHO) classification, gliomas are categorized into WHO grade I–IV. The most malignant and also most common high-grade glioma is glioblastoma multiforme (GBM). Most primary GBMs arise de novo in older patients, while secondary GBMs develop upon the progression of grade II or III gliomas; reviewed in [5]. According to the U.S. central brain tumor registry, the incidence of primary brain tumors is approximately 1 in 7,000 people per year [6,7]. Patients with high grade glioma have a very poor prognosis with a median survival time of approximately 15 months for a newly diagnosed GBM and 5–7 months for a recurrent GBM [8,9,10].

**Figure 1:**
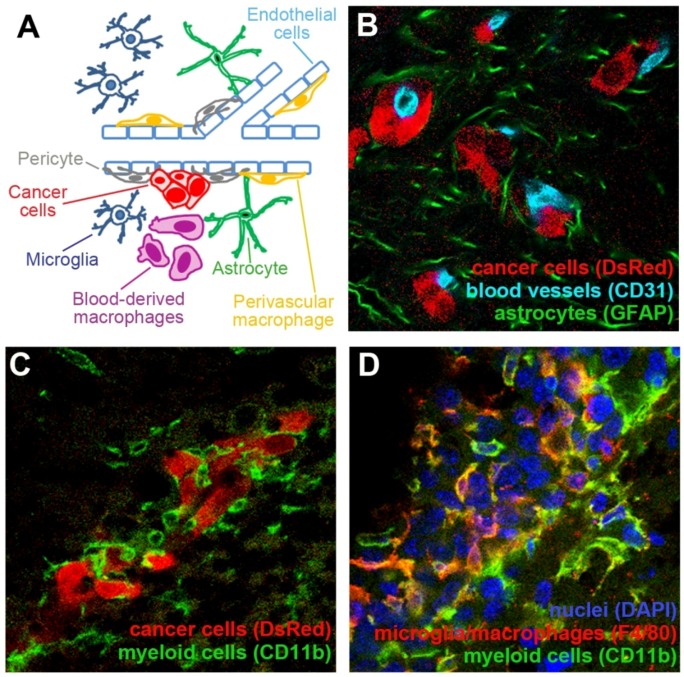
Tumor microenvironment in the brain consists of different cell types. (**A**) Schematic representation of some major cell types that constitute tumor microenvironment in the brain. (**B**) Mouse breast carcinoma cell line EO771 that stably expresses red fluorescent protein (DsRed; red) metastasized to the brain upon the administration of cancer cells into the internal carotid artery. Extravasated cancer cells remained associated with the blood vessels (light blue). The presence of cancer cells induced a strong activation of astrocytes, as demonstrated by their increased expression of GFAP (green). (**C**) Small metastatic lesions of EO771 cells (red) are surrounded by CD11b^+^ cells of myeloid origin with different morphologies (green). (**D**) A larger metastatic lesion established by EO771 cells (nuclear staining in blue) is infiltrated by CD11b^+^ myeloid cells (green). Some of these cells are macrophages/microglia as demonstrated by their positive staining for F4/80 (red).

In addition to cancer cells, tumor lesions contain a mixture of different stromal cells. This for example includes endothelial cells (EC) that constitute blood vessels, as well as inflammatory cells that infiltrate tumors from the blood stream. These stromal cells are jointly referred to as tumor microenvironment. The essential role of tumor microenvironment in cancer progression has been well documented for extracranial malignancies and recent findings indicate that tumor microenvironment may be a suitable target in anti-cancer therapies, as well as a valuable biomarker for prognostic purposes [11,12,13]. The brain provides a unique environment with paracrine growth factors that differ from most other organs [14,15]. The involvement of brain-resident and infiltrating cells in the pathology of primary and metastatic brain tumors is poorly understood. This review seeks to summarize our current understanding of interactions between tumor cells and the three cell types in the brain tumor microenvironment that have been so far most extensively studied, namely brain endothelial cells, cells of myeloid origin (e.g., microglia/macrophages), and astrocytes (Figure 1).

## 2. Interactions between Blood Vessels and Cancer Cells in the Brain

The brain is one of the most densely vascularized organs and malignant brain tumors are among the best vascularized tumors in humans. In experimental mouse models, tumors originating from the same cancer cell line display 50% higher blood vessel density during their growth in the brain as compared to the subcutaneous space (s.c.) [16,17]. In comparison to the endothelial cells in subcutaneous tissue, brain endothelial cells seem to respond differently to one of the main inducers of angiogenesis, namely vascular endothelial growth factor (VEGF). VEGF over-expression in cancer cells has been shown to increase angiogenesis in brain tumors, but not in tumors originating from the same cancer cell line growing s.c. [17,18].

Blood vessels in the brain significantly differ from the blood vessels in other organs in terms of their tightness and structure. Prominent tight junctions between brain endothelial cells and metabolic barriers strongly restrict the passage of cells and even small molecules through the blood-brain barrier (BBB). In addition to pericytes surrounding brain vessels and smooth-muscle cells that support larger vessels, brain vessels are supported by astrocyte end feet processes, which are thought to contribute to the tightness of the BBB [19,20,21].

Upon their extravasation from the blood stream into the brain parenchyma, the majority of metastatic cancer cells stay in a close proximity to blood vessels (Figure 1A,B) [22,23]. This tight association of extravasated tumor cells with the abluminal site of the vessels seems to be critical for the initiation of successful tumor growth, as non-vessel associated cells have been shown to regress [24]. Upon the initiation of brain metastases or primary brain tumors, the BBB seems to remain intact as long as the malignant lesions are smaller. In larger lesions, however, the vessels become leaky and a dissociation of pericytes and astrocytes from the vessel wall can be observed [25,26,27]. The blood-tumor barrier is affected to a variable degree within and between different primary and metastatic brain tumors [26,28]. In a recent study performed in an experimental brain metastases model, the permeability of blood vessels was increased in more than 89% of brain tumor lesions. However, despite the increased leakiness of tumor vessels as compared to the normal brain vessels, the uptake of drugs Paclitaxel and Doxorubicin by tumor lesions was lower than 15% of that found in the tissues other than brain or in peripheral metastases [28]. This suggests that although the vessel permeability in brain tumors is increased as compared to the normal brain tissue, it may still be significantly lower than in other tissues or extracranial metastases.

### 2.1. Neovascularization in Brain Tumors

Hyperdilated and proliferating blood vessels are characteristic of high-grade brain tumors and brain metastases in patients and in experimental models [25,29]. Different mechanisms of neovascularization have been described for brain tumors. These include growth of cancer cells around the pre-existing blood vessels (vessel cooption), sprouting of vessels (angiogenesis), and vasculogenesis which is a recruitment of endothelial progenitor cells (EPCs) that have been proposed to originate from different sources, including the bone marrow, the existing vasculature, or adipose tissue; reviewed in [30,31]. Another mechanism of neovascularization may include a recently reported differentiation of glioblastoma cancer stem cells to endothelial cells which then contribute to tumor vasculature [32]. Notably, these different mechanisms may co-exist within the same tumor. Experimental brain metastases as well as primary brain tumors initially grow by cooption of brain vessels [22,23,33,34] and angiogenesis is induced later on in the process of tumor progression [34]. Angiogenesis is regulated by proteases that disrupt the vascular basement membrane, angiogenic growth factors such as VEGF or Basic fibroblasts growth factor (bFGF), angiogenesis inhibitors like trombospondin-1 and angiostatin, and factors regulating the stabilizing interactions between ECs and the supporting pericytes/smooth-muscle cells like angiopoietin-1 and -2; reviewed in detail in [35,36,37,38]. Depending on the phenotype of tumor cells, the angiogenesis in brain tumors can become critical for the growth of microscopic lesions when they are not larger than 10 cells [24], or only after a prolonged cooptive growth when the tumor volume has increased significantly, resulting in hypoxia-induced VEGF expression and angiogenesis [34,39]. Upon the treatment with VEGF pathway inhibitors, primary brain tumors as well as brain metastases can switch from the angiogenic to the cooptive growth and by that evade the anti-angiogenic therapy [24,40,41,42].

During vasculogenesis, EPCs are mobilized from the bone marrow by increased concentrations of chemokines, growth factors and other soluble factors in serum, including stromal cell-derived factor-1 (SDF-1), VEGF, and granulocyte-monocyte colony stimulating factor (GM-CSF); reviewed in detail in references [43,44]. In patients with glioma, circulating EPCs (cEPCs) were found to be increasingly mobilized from the bone marrow and their mobilization correlated with increased levels of VEGF and GM-CSF in serum, as well as with a higher vessel density in tumors [45,46]. However, incorporation of EPCs into functional blood vessels in brain tumors has been controversial. Some studies investigating experimental animal models have reported a significant infiltration of EPCs into brain tumors and their differentiation into tumor endothelium, while others reported a complete lack of EPC incorporation into the newly forming vessels [47,48,49,50,51,52,53]. For example, de Palma *et al*. [48] detected an incorporation of EPCs into the mammary tumors growing s.c., but not into the glioma tumors growing in the brain. Duda *et al*. [49] demonstrated that the percentage of tumor-incorporated EPCs strongly varies with the tumor type and site, reaching 60% in a breast cancer brain metastasis model. Moreover, the number of circulating EPCs, their homing to tumors, and the contribution to tumor neovascularization in animal models have been shown to strongly increase upon certain treatments, including vascular disrupting agents and cyclophosphamide treatment at a maximum tolerable dose [54,55]. Thus, the contribution of vasculogenesis to tumor growth may depend on the tumor type and grade, the target organ, and treatment conditions.

Tumors can also increase their microvessel density by vascular remodeling. Single glioma cells migrating along the abluminal site of blood vessels and single extravasated metastatic cells have been shown to induce such vascular remodeling, resulting in a formation of vascular loops and glomeruloid bodies [56,57]. Cancer cells that are attached to the vascular basement membrane seem to pull the capillaries into the cancer cell nests, looping and coiling them up, leading to the generation of microvascular structures whose appearance resembles renal glomeruli. The molecular mechanism underlying the formation of glomeruloid bodies is not completely understood. Angiopietin-1 has been identified as one of the crucial factors in this process in brain tumors [58].

The pre-existing brain capillaries can also be multiplied by the so-called intussusceptive microvascular growth. This occurs by partitioning of the vessel lumen starting with the formation of transluminal endothelial bridge, followed by the reorganization of the endothelial lining and development of a connective tissue pillar through the vessel lumen [57]. Intussusceptive microvascular growth and vascular loop formation are much faster than sprouting angiogenesis and occur within hours from the initial contact between cancer cells and ECs.

### 2.2. Neovascularization-Independent Contribution of Endothelial Cells to the Brain Tumor Progression

It is well accepted that blood vessels promote tumor growth by supplying the nutrition and oxygen to cancer cells. In addition, tumor cells use blood vessels to spread through the brain parenchyma by migrating along the abluminal vessel site between endothelial cells and astrocyte end feet processes. This phenomenon is known as perivascular extension [59]. Furthermore, the interaction between vascular basement membrane and cancer cells in the brain can promote tumor growth via integrin engagement and the activation of downstream signaling pathways [22]. Glioma cells have been shown to pause near the vascular branch points to divide, suggesting that tumor cell division might be triggered by environmental cues from the endothelium [59].

The maintenance of the cancer stem cell (CSC) population in primary brain tumors also depends on the presence of the so-called perivascular niche [60,61]. Glioma CSCs have a higher propensity to associate with blood vessels than the tumor bulk and endothelial cells are thought to expand the CSC pool by secretion of soluble factors. For example, a recent study suggests that the secretion of nitric oxide (NO) by endothelial cells activates the Notch signaling in adjacent cancer cells and thereby enhances the CSC phenotype in glioma [62]. *Vice versa*, glioma CSCs have been shown to promote tumor angiogenesis by secreting VEGF [63].

## 3. Pericytes

Pericytes are perivascular cells that support blood vessels and promote vascular maturation [64]. Pericyte progenitor cells (PPCs) express platelet-derived growth factor receptor β (PDGFβ) and are recruited to the blood vessels by PDGFβ that is secreted by endothelial cells [65]. Mice deficient in PDGFRβ or PDGFβ expression have severely decreased pericyte coverage of blood vessels associated with increased microvascular leakage [66,67]. Moreover, depletion of pericytes through administration of anti-PDGFRβ antibody has been shown to lead to enlargement and hyperdilation of tumor vasculature in a mouse model of pancreatic cancer, accompanied by strongly increased apoptosis of endothelial cells [68]. In addition to PDGFRβ, nerve/glial antigen 2 proteoglycan (NG2) expressed on peritytes has been also shown to be crucial for their recruitment to the blood vessels, for their interaction with endothelial cells and for their maturation [69,70]. NG2 physically interacts with β1 integrin and can activate this integrin expressed on endothelial cells, promoting their morphogenesis [71]. Thus, pericytes control blood vessel stability and function through paracrine factors and through direct cell-cell contacts.

Pericytes are characterized by the expression of different pericyte markers including PDGFRβ, α-smooth muscle actin (αSMA), desmin, and NG2. None of these markers are restricted to pericytes only. Usually not all of the markers are co-expressed and their expression may be associated with different differentiation stages or different tissues. PDGFRβ is expressed on pericyte progenitors, while αSMA, desmin, and NG2 are expressed on mature pericytes. Pericytes can develop from various cell types, most commonly from the mesenchymal stem cells (MSCs) [72]. It has been shown that pericytes in tumors can originate from MSCs as well as from HSCs [68,73,74].

An excellent recent review by Winkler *et al*. [75] summarizes the role of pericytes in the central nervous system. Briefly, capilaries in the central nervous system have significantly higher pericyte coverage as compared to the peripheral tissue. Depending on their location within the brain, pericytes originate from the mesoderm-derived MSCs or neuroectoderm-derived neural crest cells. Pericytes are an essential element of the neurovascular unit. Their contribution to the functional blood-brain-barrier has been indicated for a long time and recent *in vivo* findings unequivocally confirmed these indications [76,77]. Pericyte-deficient mice have been shown to fail to down-regulate the expression of genes associated with increased vascular permeability, like for example angiopoietin-2 [77]. In addition to regulating the BBB permeability, pericytes are also involved in the regulation of cerebral blood flow and clearance of toxic cellular byproducts [57].

### The Role of MSCs and Pericytes in Brain Tumor Growth

Human MSCs have been shown to up-regulate the expression of pericyte markers desmin, αSMA, and NG2 upon the stimulation with glioma-conditioned cell culture medium, suggesting that glioma can induce the differentiation of MSCs into pericytes [78]. MSCs injected into brain tumors in mouse models have been shown to closely associate with the tumor vasculature and to also up-regulate the expression of pericyte markers [74]. The functional contribution of externally administered MSCs to tumor growth in the brain seems to be unclear with reports including no impact of engrafted MSCs on glioma growth [74], prolongation of animal survival upon the administration of MSCs in glioma model [79], as well as reduced survival of brain tumor-bearing animals [80]. These differences in experimental observations may be due to different origins of MSCs, their different administration routes, as well as specific characteristics of the analyzed animal models.

The infiltration of brain tumors by bone marrow-derived PPCs also seems to depend on the experimental model. For example, Du *et al*. [81] found that approximately 2% of bone marrow-derived cells infiltrating experimental glioma were PDGFRβ^+^ScaI^+^ PPCs. Similarly, Bababeygy *et al*. [73] reported incorporation of HSC-derived pericytes into brain tumors. In contrast to that, two studies found bone marrow-derived pericytes in mammary fat pad or s.c. tumors, while in intracranial tumors none of the bone marrow-derived cells differentiated into pericytes [47,48]. This suggests that the tumor microenvironment may co-determine the fate of bone marrow-derived progenitors. All studies used mice reconstituted with GFP-tagged bone marrow which should enable detailed tracking of bone marrow-derived cells infiltrating tumors. In the study performed by Du *et al*. [81] the recruitment of bone marrow-derived pericytes to experimental glioma strongly depended on the expression of MMP-9 in CD45^+^ vascular modulatory cells that were recruited to tumors via SDF1/CXCR4 axes. It has been suggested that VEGF released from the extracellular matrix by MMP9 mediates the recruitment of bone marrow-derived PPCs as well as EPCs to glioma, thereby promoting angiogenesis. In the absence of MMP9, tumor vessels displayed a 50% lower coverage with pericytes. Similarly, the down-regulation of MMP2 expression in experimental model of glioblastoma multiforme resulted in a 30–40% reduced pericyte coverage of blood vessels [40].

The role of pericyte—endothelial cell interaction in brain tumor growth has been recently investigated in NG2 deficient mice [69]. NG2 deficiency in the host tissue resulted in a significantly reduced tumor growth. Although these tumors showed similar microvascular density, vessel diameter and absolute numbers of pericytes as compared to tumors growing in the wild type mice, an increased number of pericytes lacked physical association with blood vessels and the pericyte coverage of vessels was strongly decreased. The tumor vessels in NG2 deficient animals were less well perfused and leaky. NG2 deficiency in the host tissue also impaired pericyte maturation and strongly reduced the deposition of collagen IV and VI in endothelial basal lamina. In line with that it has been reported that collagen VI deficiency impairs pericyte maturation and increases vessel leakiness in a brain tumor model [82]. Although the association of pericytes with blood vessels is known to be loosened in tumors as compared to normal tissue, these studies show that the pericyte-vessel interactions are still crucial for tumor growth.

## 4. Contribution of Myeloid Cells to Brain Tumors

Different cell populations of myeloid origin, characterized by the expression of cell surface marker CD11b, have been shown to infiltrate malignant brain lesions and to play a role in brain tumor progression. The described cell populations include microglia, tumor-associated macrophages (TAM), myeloid-derived suppressor cells (MDSC), Tie2-expressing monocytes (TEM), and CD11b^+^CD45^+^ vascular modulatory cells. Notably, some of these cell populations partially overlap (Figure 1C,D).

Monocytes originate in the bone marrow (BM) from a common myeloid progenitor shared with neutrophils. Blood monocytes are divided into at least two subsets: the resident monocytes (CD14^high^CD16^−^ in humans, CD11b^+^(Ly6C)Gr-1^−^CCR2^−^CX3CR1^+^ in mouse) and the inflammatory monocytes (CD14^+^CD16^+^ in human, CD11b^+^(Ly6C)Gr-1^+^CCR2^+^CX3CR1^−^ in mouse) [83,84]. Resident monocytes enter different tissues to constitutively replenish the resident tissue macrophage populations under normal conditions. Inflammatory monocytes are precursors of macrophages and dendritic cells that are recruited to the sites of inflammation. Their number drastically increases in the blood under pathologic conditions, including cancer. Macrophages are multifunctional cells and their phenotype has been shown to be modified by the local environment, in particular by the combination of different cytokines, chemokines and growth factors; reviewed in [84,85,86]. The M1/M2 polarization paradigm divides macrophages into those that are activated by the T_h_1-type cytokines IFNγ and LPS, resulting in up-regulation of nitric oxide synthase 2 (Nos2) and in pro-inflammatory phenotype (classical activation, M1 macrophages), and in macrophages activated by the T_h_2-type cytokines IL-4 and IL-13, resulting in the up-regulation of arginase 1 (Arg1) and leading to the pro-angiogenic and pro-tumoral activity (alternative activation, M2 macrophages) [87,88]. In the majority of tumors investigated so far, macrophages seem to have M2-like phenotype [87]. A recent review by Gordon proposes several additional macrophage activation and deactivation phenotypes, suggesting a high complexity and plasticity of the monocyte/macrophage cell lineage [85].

### 4.1. Macrophages and Microglia in the Central Nervous System

The CNS contains different subsets of macrophages, most prominent including the parenchymal microglia and the perivascular macrophages [89,90]. Perivascular macrophages participate in antigen-presentation at the blood-brain barrier. They have a high turnover rate and are constitutively replenished by circulating monocytes [91]. In contrast, parenchymal microglia are differentiated tissue macrophages thought to take up residency in the brain during embryonic development as fetal macrophages [92]. This view, however, has been challenged by several studies showing that monocytes infiltrate also the adult brain and differentiate into parenchymal microglia, although the monocyte turnover seems to be very slow in a healthy brain [89,90,93,94,95,96,97]. This replenishment of parenchymal microglia from the bone marrow compartment also seems to be restricted to specific brain regions [98]. Microglia can proliferate in situ and this might be the main source of microglia in adults. However, under pathologic conditions such as viral encephalitis, metachromatic leukodystrophy, and Alzheimer’s disease, infiltrating inflammatory monocytes seem to be the main source of microglia/macrophages [99,100].

Microglia are the primary immune effector cells of the CNS and are capable of generating significant immune responses. In a healthy brain, resting or ramified microglia with thin processes are distributed throughout the brain tissue. It has been suggested that upon stimulation, these resident microglia can be rapidly activated via at least two different and functionally distinct morphological states, termed activated and reactive/amoeboid microglia [89,101]. Activated microglia have a hyperdilated stellate morphology and express only Major histocompatibility complex class I (MHCI). Reactive microglia are large cells with amoeboid morphology. They express MHCI and MHCII and therefore possess increased antigen presenting capability, as well as phagocytic activity. The physiology of microglia has been recently extensively reviewed in Kettenmann *et al*. [102].

Microglia and peripheral macrophages can be distinguished by flow cytometry based on different levels of CD45 expression. Low levels of CD45 are expressed in ramified microglia, medium levels in activated/reactive microglia, and high levels in macrophages [90,103,104]. It is however difficult to reliably differentiate microglia from macrophages by immunocytochemistry. The 5D4 antibody against keratan sulfate epitope has been for example shown to detect only microglia and not macrophages in rat tissue [105,106]. Many studies used the term microglia, macrophages or microglia/macrophages to describe potentially mixed cell populations. Here we use the terms microglia and macrophages only for studies that clearly distinguished these two cell populations by flow cytometry.

### 4.2. Microglia and Macrophages in Primary and Metastatic Brain Tumors

Activated/reactive microglia and macrophages that are defined by increased expression of F4/80 (mouse) or CD68 (human) are frequently found to infiltrate primary and metastastic brain tumors in human patients and in animal models [23,107,108,109,110,111,112]. Microglia/macrophages account for 8–78% of all cells in human gliomas and 4–70% of cells in human brain metastases [113,114]. Microglia and macrophages associated with brain tumors have been shown to proliferate, which most likely contributes to their increased quantity [115,116]. However, in animal models that harbor GFP-labeled bone marrow-derived cells to allow for their tracking, a significant proportion of the glioma-associated F4/80^+^ microglia/macrophages has been shown to originate from the newly infiltrating bone marrow-derived monocytes [48,50]. Consistently, one study determined that microglia account for 13–34% and macrophages for 5–12% of viable cells in experimental gliomas [117]. Thus, the brain tumor-associated microglia/macrophages seem to be a mixed population derived from the brain-resident microglia and from the newly infiltrating monocytes.

The reaction of microglia/macrophages to cancer cells is immediate, since these cells have been found accumulating around single metastatic cancer cells that just started to extravasate into the brain from the blood stream in animal models [23]. Glioma cells secrete many factors potentially responsible for the recruitment of microglia/macrophages, including macrophage chemoattractant protein 1 and 3 (MCP-1, MCP-3), G-CSF and HGF [92,118,119,120,121]. Interestingly, microglia and macrophages have been reported to differ in their chemotactic responses to the glioma-secreted factors [122].

In patients with primary brain tumors, the number of microglia/macrophages is higher in GBM as compared to the grade II or III gliomas, and correlates with a higher vascular density [123]. The proportion of alternatively activated T_h_2-polarized CD163^+^CD204^+^ M2 microglia/macrophages with anti-inflammatory properties has also been shown to correlate with a higher tumor grade, being the highest in GBM [124].

### 4.3. Functional Role of Myeloid Cells in the Progression of Brain Tumors in Animal Models

Microglia/macrophages secrete multiple cytokines, growth factors, enzymes, and reactive oxygen species that can directly or indirectly lead to angiogenesis (e.g., VEGF), tumor proliferation (e.g., EGF), and invasion (e.g., metalloproteases) of glioma and metastatic cancer cells in the brain [89,90,108,110,125,126]. In line with that, several experimental studies suggest that microglia/macrophages contribute to tumor progression. For example, suppression of microglial/macrophage activation using minocycline resulted in a decreased optic glioma proliferation in the Nf1-deficient mouse model [107]. Another study used transgenic mice expressing thymidine kinase under the myeloid-specific CD11b promoter to test the effect of microglia/macrophages depletion on glioma growth. In this model, myeloid cells are depleted by administration of ganciclovir, a prodrug that is converted into toxic nucleotide analogues by thymidine kinase. In this study, the intra-tumoral administration of ganciclovir resulted in 70% depletion of tumor-associated microglia/macrophages and in 80% decreased tumor volume, suggesting that microglia/macrophages promote glioma growth [126].

The so called vascular modulatory cells of the myeloid lineage (CD11b^+^CD45^+^) have been shown to be recruited to glioma via the SDF-1/CXCR4 axes upon the hypoxic up-regulation of SDF-1 and HIF-1α in tumors [81]. These cells expressed MMP-9, which released matrix-bound VEGF and by that increased tumor angiogenesis. A similar molecular mechanism has been described for the pro-tumorigenic activity of CD11b^+^ myelomonocytes that are potently recruited to experimental glioma after irradiation [127]. These cells were essential for tumor re-growth after the irradiation treatment by increasing the number of functional blood vessels in a HIF-1α and SDF-1-dependent manner, thereby increasing the tumor blood flow.

Tie-2 expressing monocytes (TEM) are a subset of circulating and tumor-infiltrating monocytes that express angiopoietin receptor Tie-2 [48]. TEMs express bFGF and have been shown to account for the majority of proangiogenic activity of the tumor infiltrating myeloid cells in glioma, although they represent only 9% of the bone marrow-derived F4/80^+^ cell population [48]. Consequently, a depletion of TEMs resulted in a reduced angiogenesis and a reduced glioma growth. Recent data indicate that TEMs are derived from the resident blood monocytes and are strongly polarized towards the M2 activation state with an enhanced proangiogenic and reduced proinflammatory activity [128]. It has been further suggested that resident and inflammatory monocytes may be differentially committed to generate tumor infiltrating TEMs and TAMs, respectively [128]. Although TAMs are also polarized towards the M2 phenotype, this polarization seems to be even stronger in TEMs.

In contrast to the experimental studies described above, some studies demonstrated that myeloid cells may actually suppress brain tumor growth. Galarneau *et al*. [129] used the transgenic mice model expressing thymidine kinase under the myeloid-specific CD11b promoter. Other than in the study performed by Markovic *et al*. [126] where ganciclovir was administered directly into the tumor, Galarneau *et al*. administered ganciclovir systemically. This resulted in a ~45% depletion of tumor-associated microglia/macrophages and in 33% increased tumor volume, suggesting that microglia/macrophages inhibit glioma growth. Thus, the two studies offer seemingly conflicting results [126,129]. However, different routes of ganciclovir administration may have resulted in a depletion of distinct sub-populations of CD11b^+^ myeloid cells with different consequences for the tumor growth in the brain.

Another evidence for the anti-tumor activity of myeloid cells in the brain comes from the study performed by Kanamori *et al*. [130]. In this study macrophages were depleted through a systemic administration of macrophage-specific antibodies. This resulted in a significant increase in experimental glioma growth, again suggesting that macrophages suppress tumor growth in the brain. Lastly, an *in vitro* study demonstrated a potential tumor cytotoxicity of microglia towards lung cancer brain metastases [131]. In summary, different populations of myeloid-derived cells may exert diverse effects on the intracranial tumor growth. Moreover, the consequences of these cellular interactions may also depend on the tumor type and its molecular background.

### 4.4. Microglia/Macrophages Contribute to the Immunosuppressed Environment in the CNS

Although innate immune responses, including the recruitment and activation of peripheral macrophages and resident microglia are readily initiated within the CNS, the adaptive immune responses that involve the antigen-driven activation of lymphocytes are inhibited within the brain (reviewed in [132]). Notably, this inhibition is restricted to the brain parenchyma, as T-cell responses readily occur at other locations in the brain (e.g., ventricles, meninges). Over the recent years it has been well established that lymphocytes can enter the brain despite the intact blood-brain barrier, but their responses to antigens in the brain are directed in a different way than in other tissues; reviewed in [132].

Microglia/macrophages are thought to contribute to the immunosuppressed environment in glioma. The cytotoxicity of glioma-associated microglia/macrophages and their ability to induce effective anti-tumor T-cell responses are impaired, most likely due to the glioma-secreted immunosuppressive factors like TGF-β, IL-10, and prostaglandin E2 [117,133,134,135,136,137,138,139]. Factors secreted by glioma also down-regulate the expression of pro-inflammatory cytokine TNF-α and MHCII on activated microglia/macrophages *in vitro* and *in vivo*, and render these cells unable to present antigens to T cells [136,138,140,141]. Another mechanism by which myeloid-derived cells contribute to the immune suppression in the CNS is through the interaction between the Fas and FAS ligand (FAS-L/CD95L). Microglia rather than macrophages have been shown to be the main source of FAS-L in gliomas and FAS-L expression resulted in a reduced lymphocyte infiltration, likely due to the FAS-L-induced apoptosis of Fas-expressing T-cells [115].

Notably, in patients with GBM, circulating monocytes have also been shown to display a reduced antigen presenting capacity and a reduced capacity to differentiate into dendritic cells. In contrast, monocytes in patients with brain metastases do not seem to be altered in that regard [142]. This suggests that potential differences in the modulation of microglia/macrophage responses may exist between the primary and metastatic brain tumors.

### 4.5. Myeloid Derived Suppressor Cells

Myeloid derived suppressor cells (MDSC) are a mixed population of myeloid cells with immunosuppressive activity, characterized as CD11b^+^Gr-1^+^ cells in mouse and CD11b^+^CD14^+^CD15^+^HLA-DR^−^CD33^+^ cells in human [143,144,145]. Under pathologic conditions, this cell population drastically increases in the blood of human patients and in animal models [144,146], and accumulates in pathologic lesions including tumors. In a non-orthotopic glioma model, MDSCs have been shown to be a main source of the immunosuppressive molecule TGF-β and to inhibit T-cell activation [147]. A recent study suggested that the majority of the MDSC population may be identical with inflammatory monocytes [147,148]. MDSC respond to T_h_1 and T_h_2-type cytokines and simultaneously up-regulate the expression of Nos2 and Arg1, demonstrating that they differ from both M1 and M2 polarized macrophages, which respond only to T_h_1 or only to T_h_2-type cytokines, respectively.

## 5. Astrocytes

Astrocytes are glial cells that become activated in response to different CNS injuries. Astocyte activation is known as reactive gliosis and is characterized by cellular hypertrophy and changes in the astrocyte gene expression patterns, including the up-regulation of Glial Fibrillar Acidic Protein (GFAP) (Figure 1B). Reactive astrocytes can exert both beneficial and harmful effects on brain tissue [149,150]. After a CNS insult, reactive astrocytes form a scar tissue surrounding the damaged area and this has been shown to be essential for the local containment of inflammation to the demarcated tissue. Notably, astrocytes can also express MHC and potentially serve as antigen-presenting cells [151,152].

### The Role of Astrocytes in Brain Tumors

Reactive astrocytes characterized by the increased expression of GFAP have been frequently observed in the vicinity of primary and metastatic brain tumors in animal models, as well as in human patients [14,23,108,112,153].

*In vitro* studies demonstrated that a variety of factors secreted by astrocytes can support the growth of primary and metastatic brain tumor cells. These include neurotrophic factors such as TGF-α, CXCL12, S1P and GDNF [110]. Specifically, IL-6, TGF-β, and IGF-I secreted by astrocytes have been shown to promote the proliferation of brain-metastatic cancer cells *in vitro* [154]. IL-8, MIF and PAI-1 secreted by lung cancer cells have been shown to activate astrocytes and to induce their expression of TNF-α, IL-1β, and IL-6. In turn, these factors promoted proliferation of cancer cells [155]. Co-culturing of lung adenocarcinoma cells with an immortalized astrocyte cell line has been shown to induce ERK1/2 and Akt phosphorylation in cancer cells, suggesting increased proliferation through the activation of these particular signaling pathways [156].

Astrocytes may also contribute to the invasiveness of cancer cells in the brain by producing heparanase, an enzyme that degrades heparin sulfate proteoglycans in the extracellular matrix [157]. Heparanase expression can be up-regulated by nerve growth factor (NGF) and NGF expression in astrocytes has been shown to be stimulated in response to cancer cell-secreted factors TGF-β1, IL-1β, and bFGF [158]. Astrocytes are also thought to promote glioma invasiveness by producing pro-MMP2 and plasminogen activator (uPA). In this context glioma cells have been shown to contribute plasminogen, which was converted to plasmin by astrocyte-produced uPA. Finally, plasmin converted pro-MMP2 to the active MMP2 [159].

A recent study indicates that astrocytes may protect cancer cells in the brain from the chemotherapy-induced apoptosis by sequestering intracellular calcium known to be implicated in apoptosis. This sequestering of calcium occurred through the gap junction communication channels and required a direct cell-cell contact between cancer cells and astrocytes. The capability to protect cancer cells from apoptosis was specific for astrocytes, because direct interactions of cancer cells with fibroblasts did not show any protective effects [160].

Astrocytes have been also implicated in the immunosuppression in the CNS. Astrocytes can down-regulate the production of pro-inflammatory cytokine TNF-α by LPS-stimulated monocytes and microglia, and suppress the up-regulation of MHCII and CD80 in these cells, thereby impairing the capability of monocytes/microglia to present antigens to T-cells and to promote T-cell activation [136]. This demonstrates that, similar to glioma cells, normal astrocytes also contribute to immunosuppression. It should be noted that transformed glioma cells have been found to be more potent in immunosuppression than normal astrocytes [136]. Simillar to microglia, astrocytes have been shown to induce apoptosis in brain-infiltrating T-cells by expressing FAS-L/CD95L [161].

In summary, experimental studies indicate that astrocytes may contribute to cancer progression in the brain through a variety of different mechanisms, including the secretion of substances that promote cancer cell proliferation and invasion, suppression of adaptive immune responses through the repression of microglial activation and induction of T-cell apoptosis, and protection of cancer cells from apoptosis through direct cell-cell interactions.

## 6. Tumor Microenvironment in the Brain: Therapeutic Opportunities and Challenges

### 6.1. Targeting Angiogenesis

Primary and metastatic brain tumors mostly express high levels of VEGF and are highly vascularized [162]. This resulted in considerable efforts to target brain tumor growth with anti-angiogenic therapies. In a recent phase II clinical trial, administration of the pan-VEGF receptor tyrosine kinase inhibitor cediranib resulted in a rapid and prolonged vascular normalization in patients with recurrent glioblastoma. This led to the alleviation of vasogenic edema, which normally result in an increased intracranial pressure that is a major cause of morbidity in patients with brain tumors [163]. Similar normalization of tumor blood vessels and a decreased tumor blood volume, resulting in prolonged survival, was observed in experimental animal models of brain metastases and glioma upon the treatment with cediranib or bevacizumab, an anti-VEGF antibody [42,164,165].

However, anti-angiogenic therapies have been repeatedly shown to result in a sustained progression of primary and metastatic brain tumors in experimental models via the cooption of pre-existing blood vessels in the adjacent healthy brain parenchyma, and consequently in increased cancer cell invasion [24,40,41,42,81,164]. It has also been reported that highly angiogenic brain metastases of lung carcinoma switch to the intravascular proliferation, vessel cooption, and capillary loop formation upon the treatment with bevacizumab. Melanoma cells that usually grow by cooption in experimental brain metastases models were not affected by bevacizumab [24]. In accordance with these experimental observations, glioma patients treated with bevacizumab developed multi-focal recurrences, suggesting that the anti-angiogenic therapy led to an increased invasiveness of the recurrent tumors [166,167]. Thus, a combination of anti-angiogenic drugs and inhibitors of invasive growth may be required for glioma tretament.

Because pericytes and their interaction with the tumor vasculature have been shown to be crucial for intracranial tumor growth in animal models [69], these cells are also expected to be valuable as therapeutic targets in anti-angiogenic therapies. Inhibiting the recruitment of EPCs to tumors may also be of therapeutic value once these cells have been better characterized. Blood vessels in the brain have also been shown to be important for the expansion and maintenance of the CSC populations in glioma [60,62]. In particular, it has been shown that the endothelial cells-conditioned medium increases the glioma CSC population *in vitro*, as determined by the sphere forming assay.

An anti-angiogenic therapy using anti-VEGFR2 antibody in a combination with the cytotoxic therapy also reduced the CSC fraction in s.c. glioma xenografts [168]. If this would be the case also in an orthotopic CSC models, however, remains to be elucidated.

### 6.2. Cellular Vehicles for the Delivery of Therapeutic Agents to Brain Tumors

Different cell types that constitute tumor microenvironment in the brain have also been explored as potential cellular vehicles for the delivery of therapeutic agents to the experimental brain tumors. de Palma *et al*. [169] used genetically engineered TEMs expressing IFN-α to deliver it to brain tumors upon the homing of TEMs to the brain. This resulted in an up-regulation of IFN-inducible genes in the host compartment, inhibition of angiogenesis, and in vascular normalization. No systemic toxicities were observed, most likely due to the specific homing of TEMs to tumors and the up-regulation of Tie2 promoter-driven IFN-α expression at the tumor site.

Neural stem cells (NSCs) have also been extensively studied as delivery vehicles in glioma and brain metastases. Administration of NSCs expressing different therapeutic agents has been demonstrated to successfully inhibit brain tumor growth in animal models [170,171]. Further cellular vehicles that have been extensively studied in brain tumors are MSCs. The degree to which MSCs display glioma tropism in animal models differs between experimental studies. Some studies reported homing of MSCs to brain tumors upon their administration to distal site in the brain or even into the carotid artery [79,80], while others found that MSCs lack long-distance glioma tropism and migrate within brain tumors only upon their intratumoral administration [74,172]. These differences are likely due to different experimental models, including different species, syngeneic versus xenograft models, and different tumor types. Similar to NSCs, MSCs expressing therapeutic agents have also been shown to inhibit brain tumor growth in animal studies [79,80,173].

### 6.3. Other Potential Targets in Brain Tumor Microenvironment

The exact role of myelod cells (e.g., microglia/macrophages) in the progression of brain tumors is not clear and experimental studies offer conflicting data, suggesting that these cells may have either tumor-promoting or inhibiting characteristics. It is well possible that different subpopulations of myeloid cells play diverse roles in brain tumorigenesis. Thus, the role of these cells in brain tumors need to be further elucidated before targeting of myeloid cells can be considered for therapeutic purposes.

Several studies indicate that astrocytes may promote tumor growth in the brain as well as the resistance of brain tumors to chemotherapy. Although strategies for the depletion of astrocytes may not be feasible, targeting individual molecular pathways in astrocytes may lead to novel therapies. However, before this can be realized, further investigations into the molecular mechanisms underlying the interactions between cancer cells and astrocytes are warranted.

## 7. Conclusions

The tumor microenvironment in the brain consists of many different cell types that involve brain-resident and brain-infiltrating cells. In this context, brain endothelial cells that constitute blood vessels, microglia/macrophages, and astrocytes have received the most attention so far. However, the complex role of the tumor microenvironment in the progression of brain tumors is still poorly understood and we are just starting to understand the interactions between cancer cells and stromal cells in the brain. It has been repeatedly demonstrated that tumor microenvironment is organ-specific. It is therefore expected that orthotopic models of brain tumors will be best suited to dissect the role of different cell subpopulations in the brain tumor microenvironment in the future.

One of the current challenges in understanding the role of the tumor microenvironment is poor definition of different cell populations. For example, new phenotypes/subtypes of myeloid cells have been described in recent years, and further cellular subtypes are expected to emerge in the future. As for astrocytes, it has been shown that they differ in various brain locations and their phenotype changes upon the exposure to different stimuli. Thus, the populations of infiltrating cells like macrophages and brain-resident cells like astrocytes are heterogeneous. The conflicting reports on the contribution of myeloid cells to brain tumor growth are therefore likely due to the analysis of different subpopulations of these cells using different experimental approaches. Thus, in the future, it will be crucial to better characterize the individual sub-populations within different cell types that constitute the tumor microenvironment in the brain and to determine their specific contributions to tumor progression. While the role of bone marrow-derived tumor-infiltrating cells can be addressed to a certain extent in mouse models using bone marrow transplantations, defining the exact role of brain-resident cells like astrocytes will most likely require the use of specific transgenic animal models.

No efficient therapies for brain tumors are currently available. Therefore, targeting of the supportive tumor microenvironment in the brain for therapeutic purposes may improve therapeutic options for the patients with primary and metastatic breast cancer. Toward this goal, it is crucial to improve our knowledge of interactions between cancer cells and stromal cells in the brain. Future studies, especially those involving orthotopic *in vivo* models and analysis of human brain tumor tissue, will hopefully provide new insight into the cellular composition and function of the tumor microenvironment in the brain, and thereby define a basis for the development of novel therapeutic concepts based on the targeting of specific populations of tumor-associated cells.
